# Quantifying and manipulating the angles of light in experimental measurements of plant gas exchange

**DOI:** 10.1111/pce.14309

**Published:** 2022-03-27

**Authors:** Z. Carter Berry, Jerry Larue, Gregory R. Goldsmith

**Affiliations:** ^1^ Department of Biology Wake Forest University Winston‐Salem North Carolina USA; ^2^ Schmid College of Science and Technology Chapman University Orange California USA

**Keywords:** diffuse light, infra‐red gas analysis, integrating sphere, LEDs, photosynthesis

## Abstract

Diffuse light has been shown to alter plant leaf photosynthesis, transpiration and water‐use efficiency. Despite this, the angular distribution of light for the artificial light sources used with common gas exchange systems is unknown. Here, we quantify the angular distribution of light from common gas exchange systems and demonstrate the use of an integrating sphere for manipulating those light distributions. Among three different systems, light from a 90° angle perpendicular to the leaf surface (±5.75°) was <25% of the total light reaching the leaf surface. The integrating sphere resulted in a greater range of possible distributions from predominantly direct light (i.e., >40% of light from a 90 ± 5.75° angle perpendicular to the leaf surface) to almost entirely diffuse (i.e., light from an even distribution drawn from a nearly 0° horizontal angle to a perpendicular 90° angle). The integrating sphere can thus create light environments that more closely mimic the variation in sunlight under both clear and cloudy conditions. In turn, different proportions of diffuse light increased, decreased or did not change photosynthetic rates depending on the plant species observed. This new tool should allow the scientific community to explore new and creative questions about plant function within the context of global climate change.

## 1 INTRODUCTION

Experimental measurements of leaf gas exchange are a cornerstone of plant physiology in both basic and applied settings. The portable infra‐red gas analysers used to conduct these measurements can control different environmental factors (e.g., humidity, CO_2_ and light) known to affect plant carbon and water exchange. The ability to manipulate light conditions is of particular interest, and portable infra‐red gas analysers typically utilize artificial light sources composed of a mix of different coloured light‐emitting diodes (LEDs; at least red and blue, but often more) that allow for the precise control of both the quantity and spectral distribution of light (e.g., LI‐COR Biosciences, 2021). However, the light experienced by plants can vary not only in quantity and spectral quality, but also with respect to the angle at which that light strikes the leaf surface (often referred to as angle of incidence).

The plant physiology community generally and implicitly assumes that the angle of incidence of light emanating from artificial light sources is perpendicular to the leaf surface. This is logical insomuch as the light source sits directly over the leaf chamber similar to the sun generally occurring above a plant canopy on a clear midsummer day. Both solar zenith angle and atmospheric scattering affect the angle of incidence of sunlight, leading to a wide range of light environments experienced by plants. Despite this, information on the angle of incidence emanating from artificial light sources for gas exchange is not readily available. One of the primary companies that manufacture gas exchange equipment (LI‐COR Biosciences) has also confirmed that they have not quantified the angle of incidence for light in their systems (M. Johnson, pers. comm.). As a variable known to affect leaf gas exchange, it is important that users know the angle of incidence in their experimental setups.

Moreover, plants are frequently subject to light that is not direct (collimated), but rather a mix of direct and diffuse (scattered) light (Steven, 1977). Diffuse light occurs when aerosols (e.g., pollution or clouds) scatter the light from the sun, changing both the quantity of light reaching the leaf surface and the angle of incidence. Due to atmospheric scattering and aerosols, even the clearest day will have some proportion of light that is diffuse; these values are often at least 15% of total light and can reach as high as 30% or 40% under midday conditions (Spitters et al., 1986; Steven, 1977). There is also evidence that the annual diffuse fraction of light was 44% in 2000 (the most recent year with data), an increase of approximately 15% since 1900 (Mercado et al., 2009). Finally, plants (and leaves) occurring below the canopy, under shade houses or in greenhouses with diffusive glazing consistently experience diffuse light.

Diffuse light can have a significant effect on leaf optics and gas exchange (Berry & Goldsmith, 2020; Brodersen et al., 2008; Vogelmann, 1993; Vogelmann & Martin, 1993), with implications for ecosystem‐level carbon and water fluxes (Baguskas et al., 2021; Mercado et al., 2009; Misson et al., 2005). Therefore, we must also consider the distributions of the angles of incidence of light as a critical variable to control when conducting gas exchange measurements. The light source for gas exchange systems would ideally reflect generalized environmental conditions, as well as allow, for the manipulation of the fraction of direct compared to diffuse light to the range of values that plants experience.

Given this, we observe a major methodological gap: a variable (angle of incidence of light) known to affect leaf gas exchange (1) has neither been quantified in the most commonly used gas exchange systems, (2) nor is there a way to manipulate this variable in these systems. Here, we describe the results of a series of experiments in which we characterize the distribution of the angles of light from common artificial light sources used on portable infra‐red gas analysers. We then demonstrate the use of an integrating sphere to effectively control the distribution of the angle of light reaching the leaf surface, creating a range of possible distributions from almost entirely direct light (light from a 90° angle perpendicular to the leaf surface) to almost entirely diffuse (light from an even distribution drawn from a nearly 0° horizontal angle to a perpendicular 90° angle). Finally, we demonstrate that manipulating the distribution of angles of light has different impacts on leaf photosynthesis depending on the plant species. Our objective is to advance our understanding of, and our ability to measure, how differences in the distribution of light angles reaching leaves affect plant gas exchange.

## 2 METHODS

**Figure 1:**
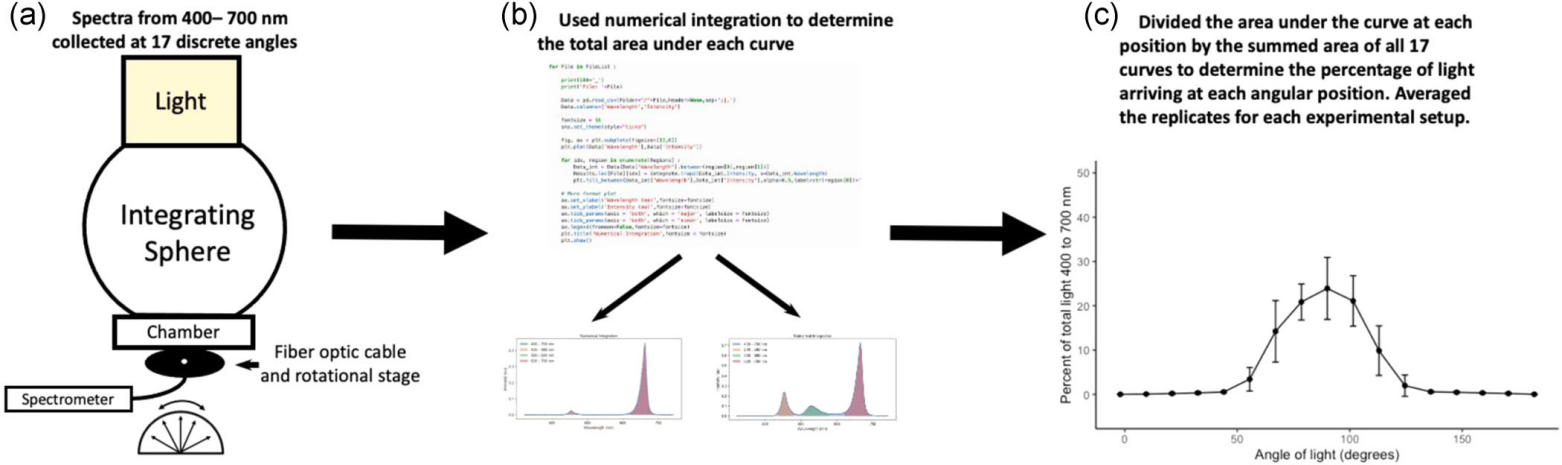
Workflow diagram from data collection of spectra to figures that quantify the angular distribution of light for different experimental setups of common portable plant leaf infra‐red gas analysers. First (a), spectra were collected with a fibre optic connected to a rotational stage and a spectrometer. The relative intensity was collected at 17 discrete angles to capture the total light of the light source. For each spectrum (b), we used numerical integration through a programming script to calculate the total area under the curve from 400 to 700 nm. Finally (c), the total area under the curve at each angular position was divided by the summed area under the curve for all 17 spectra collected for each experimental setup. For each experimental setup, 10–25 replicates were conducted and averaged  [Color figure can be viewed at wileyonlinelibrary.com]

We quantified the angular distribution of light using the small (6800‐02) and large (6800‐03) light sources from the LI‐6800 portable infra‐red gas analyser (LI‐COR Biosciences) and the small light source (6400‐02B) from the LI‐6400XT portable infra‐red gas analyser (LI‐COR Biosciences). The general workflow for our experiments is summarized in Figure 1.

### 2.1 Quantification of light angle

To assess the angular distribution of light sources that accompany different portable infra‐red gas analysers used for plant gas exchange, we measured the amount of light reaching a fibre optic cable mounted at different angles (horizontal to vertical) below the light source. The fibre optic cable was mounted to a rotational stage that allowed for it to be tilted at precise angles across a 180° field of view. The cable had a 105‐μm core diameter and a 0.1 numerical aperture, such that the angle of acceptance was 11.5° (M96L02; ThorLabs, Inc.). The fibre optic cable was mounted just below the top of the leaf chamber (where a leaf would be located) with the light source mounted on top. The fibre optic was then rotated at 11.5° increments across the field of view (horizontal‐vertical‐horizontal) to collect spectra at 17 discrete angles (a total of 184°). The fibre optic cable was connected to a CCS100 compact spectrometer (350–700 nm; ThorLabs, Inc.) and data were recorded using the ThorLabs software associated with the spectrometer.

Spectra were collected across the leaf chamber area at random positions (*n* = 10–25 positions per experimental setup). Because the rotational stage only moves along one axis, we collected spectra in two cardinal directions (i.e., front‐to‐back and left‐to‐right) to integrate the angular distribution of light in both directions. Light quantity was held constant at 1390 μmol m^−2^ s^−1^ and the distribution of wavelengths was held constant using the same proportions of LEDs in the light source (65% red, 20% blue, 10% green and 5% white).

We also assessed if changes to the ratios of LEDs used, the total intensity of light, or the method of controlling light affected the angular distribution of light. All additional tests were done using the LI‐6800 large light source. To test the effects of changing the ratio of the LEDs, we compared an LED distribution of 90% red and 10% blue to our previously conducted measurements using 65% red, 20% blue, 10% green and 5% white. The 90/10 ratio of red to blue light is referenced on the LI‐COR Biosciences website as a commonly used LED ratio (LI‐COR Biosciences, 2021). To test changes to intensity, we did additional measurements with the total photosynthetically active radiation (PAR) at 1000 μmol m^−2^ s^−1^ and compared that to our previous measurements conducted at 1390 μmol m^−2^ s^−1^. Finally, we measured the angular distribution of light using the ‘Percent’ control mode on the light‐control tab of the LI‐6800 operating system and compared this to measurements using the ‘Setpoint’ control. This was done to ensure that the percentage of each LED remained constant through our experiment. When using the ‘Percent’ control, the user is able to hold the percentage of each LED colour constant despite small fluctuations in total intensity or feedback in the system.

### 2.2 Integrating sphere

**Figure 2:**
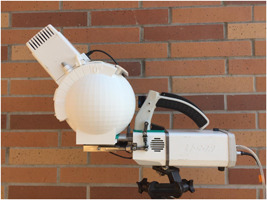
Image of the LI‐6800 large leaf chamber and light source attached to the integrating sphere. As seen, the light source is mounted at approximately 45° along the track. Photo credit: Gregory R. Goldsmith [Color figure can be viewed at wileyonlinelibrary.com]

Integrating spheres have a rich history in plant physiology and have been used to measure leaf absorptance, total radiation and leaf area (Ehleringer et al., 1976; Idle & Proctor, 1983; Serrano et al., 1997). Here, we developed an integrating sphere to provide control over the distribution of angle of light reaching the leaf surface. Specifically, we built an integrating sphere with an adjustable mount for the large light source (6800‐03) typically mounted to the large chamber (6800‐13) on the LI‐6800 (Figure 2). The sphere was 19.2 cm in internal diameter. Along one side of the chamber was an opening with rails on either side; the light source is mounted to a guide that slides along the rails between the top and the bottom, with a screw to hold it in place at the desired position. Small inserts of different sizes are slid into the rails to close that part of the sphere not covered by the light source. The bottom of the sphere has a slot that connects to the top of the large plant chamber. The inside of the sphere is uniform and smooth with no internal structures. The integrating sphere was printed in white polylactic acid (PLA; MH Build; Matterhackers Inc.) on a desktop 3D printer (Ultimaker S5). The inside of the sphere and the inserts were primed by sanding with 120 grit sandpaper and then covered with ultrawhite barium sulphate coating (Avian‐B Coating, Avian Technologies) purchased through Edmund Optics. This coating is intended to create a highly reflective surface (>97%) that effectively scatters light. The ultrawhite coating was applied as directed, which required a dilution with an alcohol solution (95% ethanol and 5% methanol) of approximately 1:1. The solution was mixed using a stir bar for 15–30 min with additional alcohol added periodically until homogenous. The solution was then evenly sprayed onto each piece with an aerosol sprayer delivered at 70 psi (Preval Sprayer, Nakoma Products). Each layer was allowed to dry until it was generally dry to the touch, typically about 5–10 min at ambient room temperature. A complete sphere, including insert and mounts, would typically require 20 layers and 200–300 ml of the total solution. The design for this sphere is patent pending (Docket 1959206.00015).

We quantified the angular distribution of light with the integrating sphere interfaced on top of the LI‐6800 large leaf chamber. Then, using the integrating sphere, we quantified the angular distribution of light for four distinct experimental setups: (1) the light source at 90° directly on top of our integrating sphere (i.e., light presumably emanating perpendicular to the leaf surface), (2) the light source at a 67.5° on the integrating sphere, (3) the light source at a 45° on the integrating sphere and (4) the light source at a 0° on the side of the integrating sphere (i.e., light presumably emanating parallel to the leaf surface). In each of these setups, the quantity of light and the proportion of LEDs in the light source were held at the same values that were used for measurements without the integrating sphere.

### 2.3 Plant photosynthesis from direct to diffuse light

To assess the effects of different distributions of angles of light on leaf photosynthesis, we collected experimental leaf gas exchange data from three different plant species. Data were collected on sun‐exposed leaves of mature trees of *Heteromeles arbutifolia* (Lindl.) M. Roem and *Citrus sinensis* (L.) Osbeck (five individuals each) at the Fullerton Arboretum (California State University at Fullerton) during February and March 2020. Data on *Persea americana* Mill. (eight individuals) were collected on sun‐exposed leaves of potted trees (approximately 2 m tall) on the campus of Chapman University from June to September 2020. Instantaneous gas exchange was quantified on leaves in five different distributions of light using the integrating sphere. The sphere was mounted to the LI‐6800 as described above. A fully expanded, mature leaf was placed in the chamber and allowed to stabilize under the following chamber conditions: total PAR of 1295 μmol m^−2^ s^−1^, temperature of 27°C, a CO_2_ of 410 μmol mol^−1^, a relative humidity of 50%, fan speed of 10 000 rpm, and a flow rate ranging from 500 to 1000 μmol s^−1^. Each leaf was first measured with the light source at one of the endpoints of the integrating sphere (0° or 90°, alternating which was first). Once the measurement was recorded, the light source was moved to the next position on the sphere and again allowed to stabilize. This process was repeated at all five light positions. Relationships between photosynthesis and the angle of the integrating sphere were analysed using linear regression.

### 2.4 Data processing

To determine the total quantity of light for each spectrum, we integrated the area under the curve from 400 to 700 nm, the wavelengths commonly referred to as PAR. We did this using numerical integration via the trapezoidal rule, where a series of trapezoidal areas are created under the curve and then summed to determine the total area. A trapezoid was computed between each data point resulting in 3647 distinct trapezoids for our data set. The numerical integration was computed using a Python script and processed in JupyterLab v2.2.6 (Project Jupyter, Worldwide). The total integrated area was summed from each of the 17 distinct curves created for each replicate. The per cent of light arriving at each angle was determined as the amount of light at that angle divided by the total summed value. The 10–25 replicates for each experimental setup were averaged to create figures that demonstrate the percentage of PAR arriving as a function of angle of light. Using the percentage of PAR instead of raw intensity values allows us to more easily compare across curves from different experimental settings. At specific angles of interest, two‐sample *t*‐tests were run to compare differences in spectra. All analyses and figures were made in R Studio v1.4.110 (R Studio) using R v4.0.3 (R Foundation for Statistical Computing).

## 3 RESULTS

### 3.1 Angular light distribution of traditional gas exchange setups

**Figure 3:**
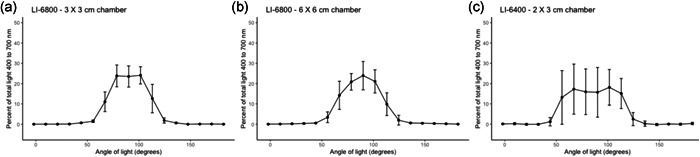
The percentage of light (400–700 nm) arriving at the leaf chamber as a function of the angular distribution of light. Data are shown for three commonly used gas exchange chambers; the LI‐6800 3 × 3 cm^2^ chamber (a; “small leaf chamber”), the LI‐6800 6 × 6 cm^2^ chamber (b; “large leaf chamber”) and the LI‐6400XT 2 × 3 cm^2^ chamber (c). Each panel had the light source in its traditional position mounted directly above the leaf chamber. Data represent means and one standard deviation

We tested the angular distribution of light with the LI‐6800 small and large leaf chambers and the LI‐6400 small leaf chamber (Figure 3 and Table 1). For all results, we refer to 90° as the position where the light is positioned directly perpendicular to the leaf surface The LI‐6800 small and large leaf chambers produced similar light angle distributions (Figure 3a,b) with 23.5 ± 5.2% and 23.9 ± 7.0% of light coming from 90 ± 5.75° (*t* = 0.21, *d.f*. = 20.5, *p* = 0.84). Both chambers had dramatic declines in the quantity of light beginning around 28.75° from perpendicular in either direction and very little light (<9% total) came from other angles. The LI‐6400 small chamber had a similar, albeit slightly broader, distribution with much greater variation at each position (Figure 3c and Table 1). This chamber had 15.7 ± 12.2% of light coming from 90 ± 5.75° which, while broader, was not significantly different from either LI‐6800 chamber (with LI‐6800 large chamber: *t* = 2.02, *d.f*. = 11.05, *p* = 0.07; with LI‐6800 small chamber: *t* = 1.85, *d.f*. = 12.13, *p* = 0.09). Light was much more evenly distributed through 90 ± 40.25°, ranging from 10% to 20% at each position. At each angle, the standard deviation reflects the variation in the angular distribution of light across different locations within the leaf chamber. The particularly high variance for the LI‐6400 small chamber was due to greater spatial variability (at different positions in the chamber) in light angle distribution.

**Table 1:** The percentage of light arriving at the leaf chamber as predominantly direct light (90°—perpendicular to leaf surface)

Light setup	Percent arriving at 90 ± 5.75°	Percent arriving at 90 ± 17.25°
LI‐6800 3 × 3 cm^2^ chamber	23.5 ± 5.2	71.4 ± 8.6
LI‐6800 6 × 6 cm^2^ chamber	23.9 ± 7.0	65.9 ± 9.9
LI‐6400 2 × 3 cm^2^ chamber	15.7 ± 12.2	49.8 ± 18.8
Sphere 90°	43.3 ± 5.4	77.0 ± 15.4
Sphere 67.5°	7.8 ± 3.2	37.4 ± 18.8
Sphere 45°	6.7 ± 3.2	23.0 ± 5.5
Sphere 0°	9.9 ± 0.5	29.9 ± 0.8

*Note*: Data are shown as the percentage of total light arriving at 90 ± 5.75° (11.5° angle of acceptance) and arriving at 90 ± 17.25° (34.5° angle of acceptance). The percentages shown represent how much of total light (from all angles 0° to 180°) was arriving from the 90° position. Data represent means and one standard deviation.

We also tested if changes to the ratios of LEDs used, the total intensity of light, or the method of controlling light affected angular distribution of light. None of these changes affected the angular distribution of light (Figures S1 and S2). Specifically, when we changed the ratio of LEDs from 65% red, 20% blue, 10% green and 5% white to 90% red and 10% blue, the percentage of light arriving at 90 ± 17.25° was a nonsignificant change from 65.9 ± 9.9% to 60.7 ± 5.9% (*t* = 1.11, *d.f*. = 26.83, *p* = 0.28). When we then tested a change in intensity by lowering the PAR from 1390 to 1000 μmol m^−2^ s^−1^, the percentage of light arriving at 90 ± 17.25° was 61.8 ± 9.0% (Figure S1; *t* = 0.20, *d.f*. = 14.57, *p* = 0.85). Finally, we tested two different methods to control light intensity and LED ratios on the LI‐6800 and again found no change to the angular distribution of light. The percentage of light arriving at 90 ± 17.25° was 65.5 ± 9.9% when run by controlling light as a ‘Setpoint’ and 63.7 ± 9.9% when controlling light as a ‘Percentage’ (Figure S2; *t* = −0.27, *d.f*. = 12.53, *p* = 0.79).

### 3.2 Angular light distribution with the integrating sphere

**Figure 4:**
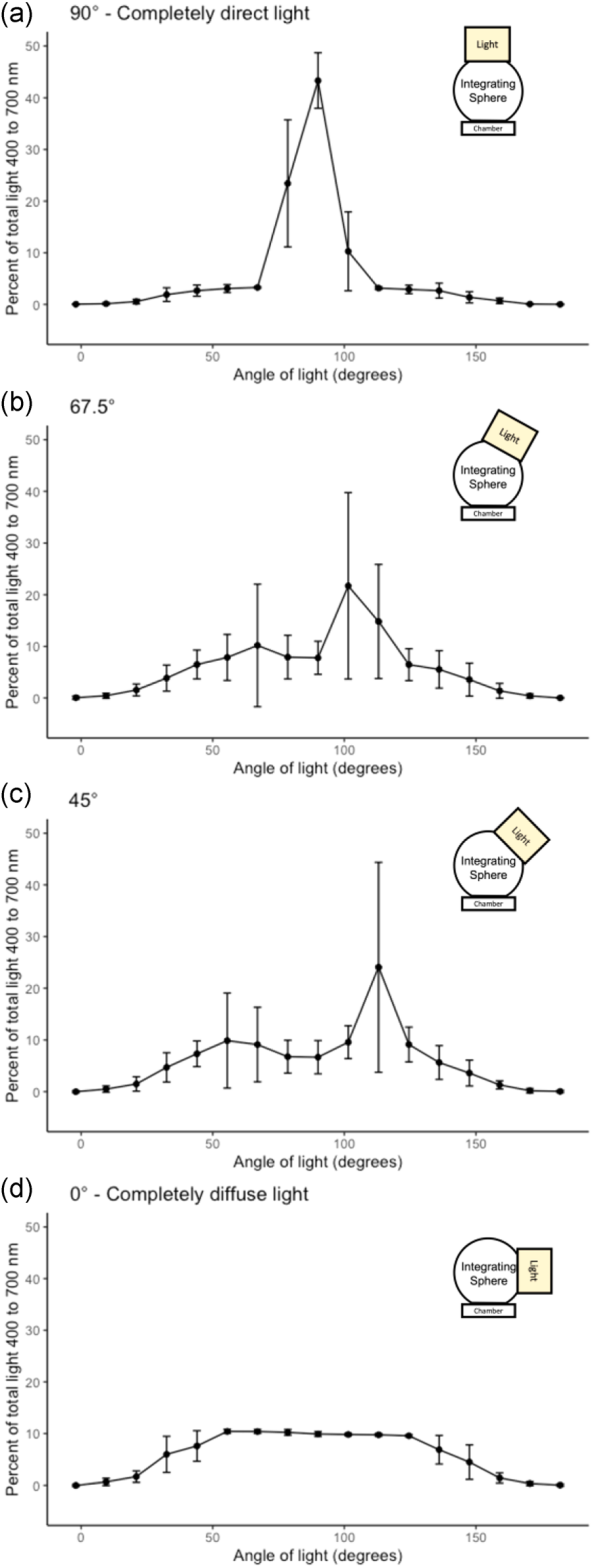
The percentage of light (400–700 nm) arriving at the leaf chamber as a function of the angular distribution of light with an integrating sphere mounted to the top of the chamber. Each panel represents the light source mounted at a different position along the track on the side including at 90° (a), 67.5° (b), 45° (c) and 0° (d). The diagram in the corner of each figure is a visual representation of the experimental setup. Data represent means and one standard deviation [Color figure can be viewed at wileyonlinelibrary.com]

We found that the integrating sphere alters the distribution of light from the LI‐6800 light source by both making light more direct when mounted directly on top of the sphere (90°) and more diffuse when the light is mounted on the side of the sphere (0°) (Figure 4). For the control (no integrating sphere on the LI‐6800 large leaf chamber), the per cent of light arriving at 90 ± 5.75° was 23.9 ± 7.0% and the per cent of light arriving at 90 ± 17.25° was 65.9 ± 9.9%. With the light mounted directly on top of the sphere, these percentages increased significantly to 43.3 ± 5.4% and 77.0 ± 15.4%, respectively (*t* = 12.00, *d.f*. = 44.90, *p* < 0.001). Conversely, when the light is mounted on the side of the sphere (0° or 180°), these percentages were reduced to only 9.9 ± 0.5% and 29.9 ± 0.8%, respectively (compared to the control: *t* = −10.85, *d.f*. = 24.56, *p* < 0.001). The intermediate positions on the sphere provide intermediate percentages of predominantly direct light, allowing the user to manipulate the amount of diffuse light reaching the leaf surface by rotating the light source around the outside of the integrating sphere.

### 3.3 Photosynthetic response to different distributions of angles of light

**Figure 5:**
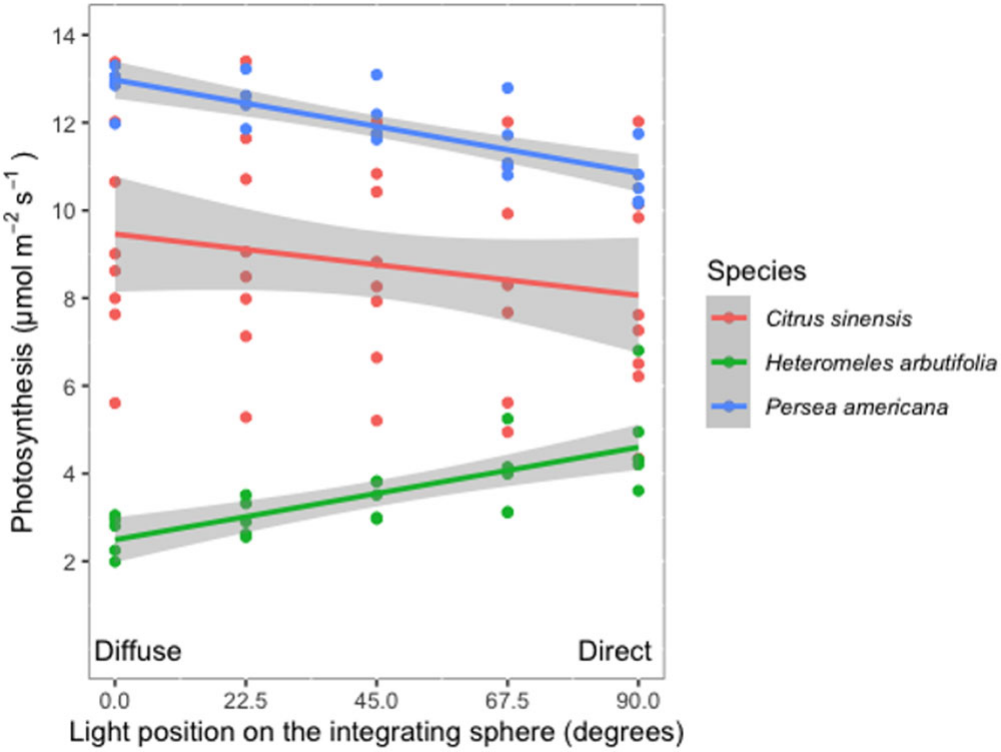
Relationship between photosynthesis and the position of the light source on the integrating sphere for three plant species. Each light position corresponds to a distinct light environment ranging from predominantly direct (0°) to predominantly diffuse (90°). Lines represent linear regressions and 95% confidence intervals (*n* = 5 individuals each for *Citrus sinensis* and *Heteromeles arbutifolia*, eight individuals of *Persea americana*) [Color figure can be viewed at wileyonlinelibrary.com]

To assess the effects of different distributions of angles of light on leaf photosynthesis, we collected experimental leaf gas exchange data using the integrating sphere on three different plant species (Figure 5). We observed distinct responses in each species, including a significant increase in photosynthetic rates with increasing proportion of diffuse light in *C. sinensis* (slope = −0.024, *p* < 0.0001), a significant decrease in photosynthetic rates with increasing proportion of diffuse light in *H. arbutifolia* (slope = 0.023, *p* < 0.0001), and no significant change in photosynthetic rates with change in the proportion of diffuse light in *P. americana* (slope = −0.0155, *p* = 0.20).

## 4 DISCUSSION

Our advances in understanding how carbon and water move across leaf surfaces have relied heavily on leaf‐level gas exchange systems for more than 50 years and now constitutes thousands of research papers annually. These gas exchange systems have led to revolutionary advances in our understanding of plant function in both basic (e.g., terrestrial carbon cycling) and applied contexts (e.g., crop productivity). These same instruments also rely heavily on light sources that are assumed to approximate characteristics of light from the sun travelling through our atmosphere and arriving at the leaf surface. However, practically all of these measurements have been taken while ignoring a key characteristic of solar radiation: the angle of light arriving at the leaf. Recent work has demonstrated that the distribution of light angles directly impacts leaf physiology (Figure 5; Baguskas et al., 2021; Berry & Goldsmith, 2020; Brodersen et al., 2008). However, until now, we were unable to control this characteristic of light. We quantified the angular distribution of light produced by common gas exchange analysers and demonstrated how adding an integrating sphere to the setup can allow for reliable manipulation of the angles of light hitting the leaf surface. Our vision is that this resource, combined with clear evidence that diffuse light alters plant function, will inspire new research directions for plant researchers.

### 4.1 Assessing angular distributions of light

The integrating sphere in this study was able to alter the angular distribution of light from light that was more direct (Figure 4a) to more diffuse (Figure 4d) than that available with the standard light source alone. This range facilitates measurements that more closely mimic the angular distribution of light from the sun. Under clear skies conditions, the proportion of diffuse light varies as a function of scattering by particles or aerosols in the atmosphere and ranges from 15% to 40% of total light under midday, clear‐sky conditions (Spitters et al., 1986; Steven, 1977). This was further supported by our own measurements where 14%–20% of midday light under clear skies arrived as diffuse light in Winston‐Salem, North Carolina during the summer of 2021 (Figure S3). The 90° position on the integrating sphere provided a similar fraction of diffuse light. Moreover, the angular distribution of light from the sun acts as a more leptokurtic distribution where data are more concentrated around the mean with sharp reductions as you move to higher angles of light (Perez et al., 1993). The integrating sphere also creates this leptokurtic curve shape, whereas the light sources we tested all create a more normal distribution.

Similarly, the integrating sphere creates a more realistic light environment for diffuse light conditions, which commonly occur with cloud cover or high atmospheric pollution. Our data demonstrated an equal percentage of light arriving at each angle in the middle 80.5° (9.5%–10.4% at each angle) followed by a gradual reduction at other angles. In overcast or cloudy conditions, nearly all light is arriving as diffuse under the conditions described above (Spitters et al., 1986; Steven, 1977). But the dynamic movement of clouds, combined with the changing zenith angle of the sun, leads to angular distributions of light that are constantly changing and somewhere in between the endpoints. The integrating sphere allows for more realistic representations of this dynamic light environment when carrying out experiments.

### 4.2 Implications for plant biology

There is a growing body of research that suggests that plant leaf gas exchange varies as a function of diffuse light (Berry & Goldsmith, 2020; Brodersen et al., 2008; Earles et al., 2017; Hughes et al., 2015; Urban et al., 2014; Vogelmann, 1993; Vogelmann & Martin, 1993). The directionality and magnitude of these changes vary from a 20% reduction to a 100% increase in photosynthesis under diffuse light. To date, epidermal lensing (Vogelmann, 1993), light transmittance mediated by leaf anatomy (Brodersen & Vogelmann, 2007; Brodersen & Vogelmann, 2010; Earles et al., 2017, efficiency or vertical distribution of chlorophyll (Hogewoning et al., 2012; Oguchi et al., 2011), or changes to CO_2_ availability from stomatal responses have all been suggested as plausible explanations. Presenting the integrating sphere in this technical report is a critical first step in elucidating which of these many competing hypotheses drives variation across species. While the mechanism remains unresolved, we add data (Figure 5) demonstrating (1) three distinct responses among three different species and (2) that intermediate proportions of diffuse light lead to intermediate rates of photosynthesis. The integrating sphere combined with these results open opportunities for developing relationships between photosynthesis and different proportions of diffuse light.

By quantifying the angular distribution of light of gas exchange systems and providing a new tool that allows for manipulation of this variable, we provide the opportunity to explore these observations and to open other new directions for research. Most fundamentally, we need to understand how plant gas exchange varies in response to diffuse light across species and given different environmental contexts. Uncovering the mechanism that explains these varied responses across species will be critical to our understanding of plant functional biology. In addition, no studies have yet explored how changes to diffuse light gas exchange change as a function of changes to other environmental variables such as CO_2_ concentration, moisture availability (soil or atmospheric), temperatures or even the spectral distribution of light. These interactions will become critical to our understanding of plant function as global climate continues to change. This includes the effects of new cloud regimes, changing aerosol patterns, and the potential for atmospheric geoengineering to alter the fraction of diffuse light and have concomitant impacts on plant function. Finally, leaf‐level observations will need to be reconciled with ecosystem measurements demonstrating changes in primary productivity under diffuse light conditions (e.g., Alton et al., 2007; Cheng et al., 2015; Gu et al., 2002; Mercado et al., 2009; Roderick et al., 2001; Williams et al., 2014).

### 4.3 Conclusions

Measurements of plant gas exchange are ubiquitous and fundamental to our understanding of plant function; the community has made tens of thousands of observations of plant leaf gas exchange since portable infra‐red gas analysers became commonly available. In turn, it has also developed the ability to carefully control environmental parameters to which gas exchange may vary, including the amount and spectral distribution of light. We have quantified the angular distribution of light in common portable infra‐red gas analysers and developed a complementary tool that will allow for more reliable control of this variable. Determining the extent and explanations of the response to the angular distribution of light has implications for all measurements that rely on our understanding plant gas exchange.

## CONFLICTS OF INTEREST

The authors declare no conflicts of interest.

## SUMMARY STATEMENT

Diffuse light alters plant gas exchange but we cannot manipulate this variable with current instruments. We quantified the angular fraction of light in common gas exchange instruments and created a new tool that allow us to manipulate this critical variable with significant effects on plant gas exchange.

## Supporting information

Supporting information.

## Data Availability

Data will be made available upon reasonable request to the corresponding author.
