# Supplementary material for: Quantifying and manipulating the angles of light in experimental measurements of plant gas exchange
Source: Plant Cell Environ. 2022 Mar 27;45(6):1954–61. doi: 10.1111/pce.14309 (PMC9314070; doi:10.1111/pce.14309)
Supplement: Supplementary file 1 — Supporting information. [file PCE-45-1954-s001.docx]

Supplementary Material


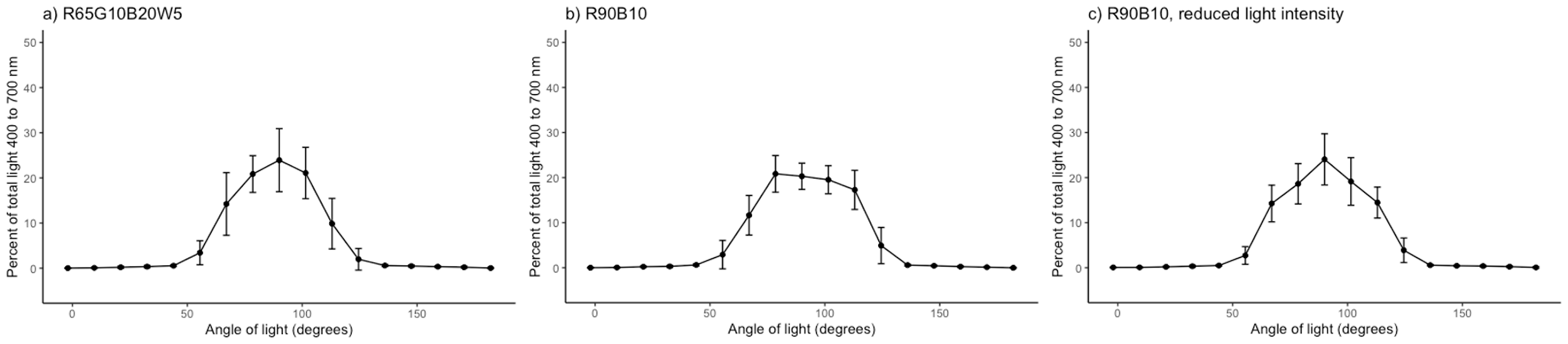


**Figure S1.** The percentage of light (400 to 700 nm) arriving at the leaf chamber as a function of the angular distribution of light with the LI-6800 large leaf chamber. These panels show tests where we changed the ratio of LEDs in the light head and the light intensity. Panel (a) shows the LED ratio of 65 % red, 20 % blue, 10 % green, and 5 % white at 1390 μmol m^-2^ s^-1^ PAR. Panel (b) shows the same PAR level but with a ratio of 90 % red, 10 % blue. This ratio of LEDs is a common ratio used for gas exchange measurements. Panel (c) shows the sale LED ratio as panel (b) but with the PAR level at 1000 μmol m^-2^ s^-1^. Data represent means and one standard deviation.


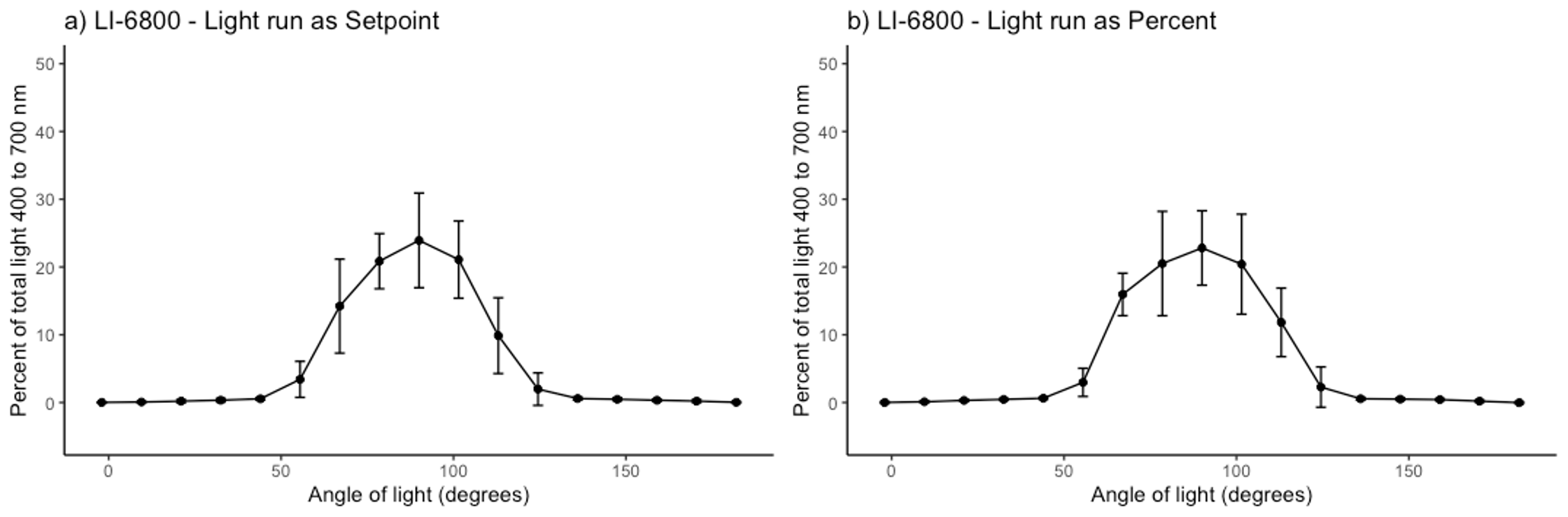


**Figure S2.** The percentage of light (400 to 700 nm) arriving at the leaf chamber as a function of the angular distribution of light. Panel (a) represents controlling the intensity and ratio of LEDs by using the “Setpoint” feature on the LI-6800. Panel (b) uses the “Percentage” feature. This test was done after conversations with colleagues at LI-COR Biosciences indicating that the two different methods could affect the quality and quantity of light (M. Johnson and D. Lynch, pers. comm.). Both experimental setups held the LED ratio of 65 % red, 20 % blue, 10 % green, and 5 % white and the light intensity at 1390 μmol m^-2^ s^-1^ PAR. Data represent means and one standard deviation.


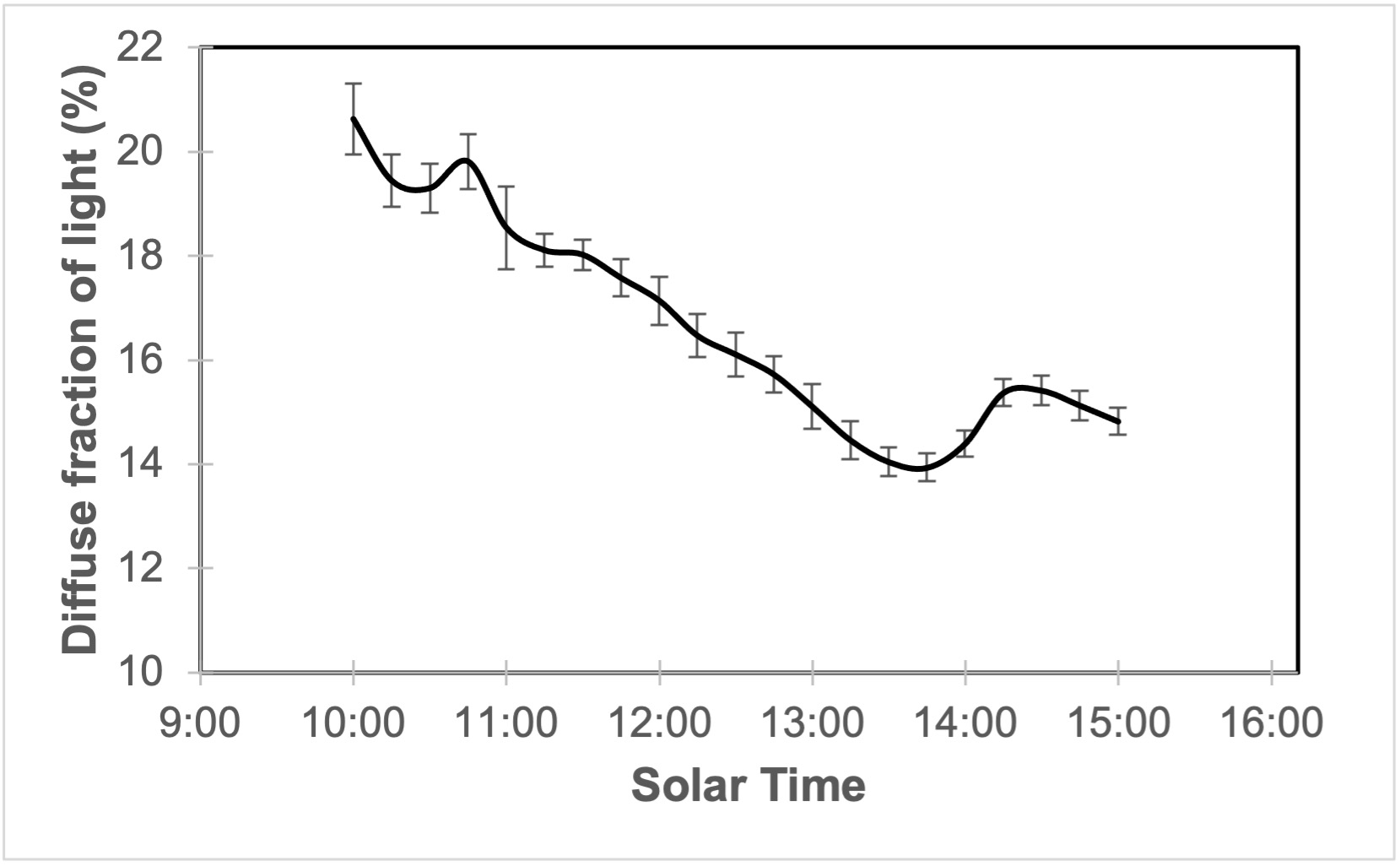


**Figure S3.** The diffuse fraction of light in Winston-Salem, North Carolina during seven consecutive clear days in the summer of 2021 (July 25 – 31, 2021). Midday (10:00 – 15:00) photosynthetically active radiation (PAR) was measured using a BF5 sunshine sensor (Delta-T Devices, Cambridge, United Kingdom), which determines the fraction of PAR arriving as direct and diffuse. These values were used to determine the percent fraction of PAR that was diffuse at each time point. The values over this period range from 14 % to 20 %, similar to other measurements of diffuse fraction of light during clear sky conditions.
